# MicroRNA-145 Mediates Steroid-Induced Necrosis of the Femoral Head by Targeting the OPG/RANK/RANKL Signaling Pathway

**DOI:** 10.1371/journal.pone.0159805

**Published:** 2016-07-26

**Authors:** Ji-Jun Zhao, Zhao-Feng Wu, Ling Wang, De-Hong Feng, Li Cheng

**Affiliations:** Department of Orthopedics, Wuxi People’s Hospital, Wuxi 214000, China; Nanjing Medical University, CHINA

## Abstract

**Objective:**

To investigate the role of microRNA-145 (miR-145) in steroid-induced necrosis of the femoral head (SINFH) by evaluating its effects on the OPG/RANK/RANKL signaling pathway.

**Methods:**

A rat model of SINFH was constructed via injection of the lentiviral vector pLV-shRNA-miR-145. Pathological observation was performed via tartrate-resistant acid phosphatase (TRAP) staining, and serum OPG levels were detected by ELISA. The mRNA expression levels of miR-145, OPG, RANK and RANKL in THP-1 cells were assessed by RT-PCR, and the protein expression levels of OPG, RANK and RANKL were assessed by western blotting.

**Results:**

The expression of miR-145 in the lentivirus-mediated miR-145 group was significantly up-regulated compared with that in the control and normal groups (both *P* < 0.01). Serum OPG levels were decreased in SINFH rats compared with control and normal rats. The mRNA and protein expression levels of OPG in THP-1 cells decreased after transfection (all *P* < 0.05). By contrast, the mRNA and protein expression levels of RANK and RANKL in THP-1 cells increased after transfection (all *P* < 0.05). After transfection of 293T cells with an miR-145 overexpression vector, miR-145 expression in 293T cells increased significantly, while OPG mRNA and protein expression decreased significantly (all *P* < 0.05).

**Conclusion:**

MiR-145 plays a role in the occurrence of SINFH by targeting the OPG/RANK/RANKL signaling pathway.

## Introduction

Osteonecrosis (ON) of the femoral head is a potentially debilitating disease that frequently affects young people [1, 2]. The clinical course of ON is unpredictable, but radiography has enabled clinicians to identify small, stable and potentially curable osteonecrotic lesions, which may not progress or cause significant joint damage. However, large lesions, including asymptomatic lesions, may cause femoral head collapse, fracture pain and secondary osteoarthritis [3–5]. Moreover, steroid-induced necrosis of the femoral head (SINFH), which is induced by high doses and/or long-term administration of steroid hormones, is one of the most serious complications of steroid administration [6–8]. Fatty marrow is a well-established early histological manifestation of SINFH *in vivo* [6]. It has been reported that SINFH occurs in patients who have received corticosteroid treatment for underlying diseases, such as systemic lupus erythematosus, nephrotic syndrome and renal transplantation [9]. Additionally, various pathophysiological mechanisms have been proposed to explain the occurrence and manifestations of this disease, including fat emboli, microfractures, microvascular tamponade, and retrograde embolization of marrow fat [1]. There have been many reports of high early failure rates in patients with steroid-induced ON, suggesting that the prognosis of SINFH is poor even after surgical treatment [9, 10]. Therefore, an effective biomarker for diagnosing and treating SINFH is urgently needed.

MicroRNAs (miRNAs) are a class of small regulatory non-coding RNAs with a length of approximately 22 bp that mediate the silencing of post-transcriptional gene expressionthrough recognition of specific mRNA sequences [11–13]. Accumulating evidence indicates that miRNAs play significant roles in various human cancers by modulating several biological and pathologic processes, such as cell differentiation, growth, proliferation, and apoptosis, as well as tumor angiogenesis, invasion and metastasis [14–16]. Therefore, miRNAs may function as novel biomarkers of disease and tools for guiding clinical therapy. Abnormal miRNA expression has been reported to influence the development of bone dysfunction [17]. The most well-studied miRNA associated with SINFH is microRNA (miR)-145, a short RNA molecule in humans encoded by the *MIR145* gene that has been found to be downregulated in hormone-NO patients, based on the results of miRNA chip assays performed by Wei *et al*. and Seeliger *et al*. [17–19]. Furthermore, Götte *et al*. reported that experimental miR-145 overexpression inhibits the actin bundling protein fascin and the junctional cell adhesion molecule JAM-A by targeting the osteoprotegerin (OPG)/receptor activator of nuclear factor-κ B (RANK)/receptor activator of nuclear factor-κ B ligand (RANKL) pathway, which is associated with bone metabolism [20,21]. Additionally, Yuan *et al*. determined that thousands of miRNAs, such as miR-21, miR-17, and miR-92a, are up-regulated, whereas others, such as miR-205 and miR-145, are downregulated in the setting of femoral head repair using high-throughput gene chip technology [22]. The OPG/RANK/RANKL pathway reportedly not only regulates the formation of multinucleated osteoclast cells but also mediates their activity and survival during normal bone remodeling [23]. Theoleyre *et al*. demonstrated that the molecular triad OPG/RANK/RANKL is involved in the orchestration of pathophysiological bone remodeling [24]. Glucocorticoid-induced femoral head necrosis can be prevented by antagonizing glucocorticoid-induced changes in OPG and RANKL expression [25]. However, the relationship among miR-145, SINFH and the OPG/RANK/RANKL pathway remains uncertain. Therefore, present study aimed to determine the potential role of miR-145 in SINFH and the relationship between miR-145 and the OPG/RANK/RANKL signaling pathway in SINFH to highlight the mechanisms underlying the effects of miR-145 in SINFH progression.

## Materials and Methods

### Ethics statement

A total of 28 adult male Sprague Dawley (SD) rats were provided by the Laboratory Animal Center of Wuxi People’s Hospital, and this study was approved by the Wuxi People’s Hospital Ethics Committee.

### Lentiviral vector construction

According to the rat Rno-miR-145 (MIMAT0000851) gene sequence in the miBase database (5’-GUCCAGUUUUCCCAGGAAUCCCU-3’), a pair of oligonucleotide chains containing the miR-145 sequence and the reverse complementary miR-145 sequence, which can form an shRNA precursor sequence containing a stem-loop structure after annealing, were designed. Primers were synthesized by Sangon Biotech Company (Shanghai, China). The pLV-shRNA lentiviral expression vector was generated via T4 DNA ligase, and an unrelated negative control sequence was established in the same manner (sequence: 5’-UUCUCCGAACGUGUCACGUTT-3’).

### Lentivirus packaging and titer determination

HEK-293T cells (5 × 10^4^; ATCC, Maryland, USA) were seeded in a 6-well cell culture plate in Dulbecco's modified Eagle medium (DMEM) and incubated at 37°C with 5% CO_2_ for 24 h. When the cells reached 80%~90% confluence, pLV-shRNA plasmids were transfected into them using Lipo2000. After 48 h of transfection, the cell supernatant was collected for titer determination. Gradient dilution was performed for the supernatant using phosphate-buffered saline (PBS) at ratios of 10^−1^~10^−6^. Three wells were used for each gradient, and 50 μl of lentiviral diluent was placed in each well for infection. After 48 h of infection, we recorded the number of infected fluorescent cells at the dilution gradient where the ratio of green fluorescent protein (GFP)^+^ cells was approximately 10%, and mean values were calculated. Lentiviral titers were calculated according to the following formula (BT = TU/ml): TU/ul = (P × N/100 × V) × 1/DF (P = number of GFP^+^ cells, N = 10^5^, V = volume of lentiviral diluent = 50 μl, DF = dilution factor).

### Construction of a rat model of SINFH and grouping

A total of 28 adult male Sprague Dawley (SD) rats (clean-grade; age, 2 months; body weight, 220~260 g) were provided by the Laboratory Animal Center of Wuxi People’s Hospital. Additionally, the body weight of male rats was larger than that of female rats, which contributes to more convenient operation in sampling; therefore, all selected SD rats for this study were male [26]. All rats were maintained in cages (485 mm × 350 mm × 200 mm) in groups of 4 at a temperature of 20–24°C, a humidity of 55–60%, an airflow velocity of 0.1~0.2 m/s, a work-illumination of 15~300 lx, an animal illumination of 15~20 lx, a 12-h diurnal cycle under artificial illumination, a ventilation frequency of 10~20 /h, an air cleanliness level of 10000, afalling bacteria count of ≤ 3/dish and a noise level ≤ 60 db and were allowed free access to food and drinking water. The rats were randomly allocated into an experimental group (20 rats) and a normal group (8 rats). The rats in the experimental group received an intramuscular injection of 10 mg/kg dexamethasone sodium phosphate (Tiankang Pharmaceutical Company, Anhui, China). After the last injection, 4 rats in the experimental group were anesthetized with intramuscular injections of 1% thiopental sodium (20 mg/kg) and euthanized with in vitro injections of air into their cardiac chambers. Bilateral intact femurs (including the hip joint) were obtained for naked-eye observation and photographing before being split into smaller pieces, fixed in 4% formaldehyde solution and decalcified in 10% ethylenediamine tetracetic acid (EDTA) solution for 2 months, during which the EDTA solution was replaced once every 3 d. After complete decalcification, the tissue pieces were washed under running water, cleared in xylene twice, embedded in paraffin wax, and cut into 6 μm sections (LEICA RM 2025). Then, the remaining 16 rats in the experimental group were divided into a lentiviral treatment group (treated with tail vein injections of pLV-shRNA-miR-145) and a lentiviral control group (treated with tail vein injections of pLV-shRNA-miR-control) after pathological confirmation of SINFH, with each group comprising 8 rats. The rats in the normal group were intramuscularly injected with the same volume of physiological saline. Injections were performed once a week for a total of 10 weeks.

### Animal sampling and specimen processing

Rat serum was collected from the inferior vena cava for OPG concentration determination. After the rats were anesthetized with intramuscular injections of 1% thiopental sodium (20 mg/kg) and euthanized with in vitro injections of air into their cardiac chambers, their left and the right femoral heads were collected, and the surrounding soft tissues were removed. Then, the femoral heads were fixed in formaldehyde solution for 24h before being decalcified, dehydrated in an ethanol gradient, treated with xylene, embedded in paraffin, and stained with hematoxylin-eosin (HE). The femoral heads were subsequently photographed and analyzed to calculate thetrabecular area percentage and the trabecular bone width within the femoral head unit area and the number of vacuolar lacunae per high-power microscopic field.

### Enzyme-linked immunosorbent assay (ELISA)

Serum OPG concentrations were determined in accordance with the specifications of an ELISA kit (Bender MedSystems, Vienna, Austria). Rat serum samples from each group, as well as a standard sample (50 ul), were added to 50 μl of assay diluent RD1-21 buffer and incubated at room temperature for 2 h. After the plate was washed with wash buffer, 100 μl of rat OPG conjugate was added to each well, and the samples were treated in a shaking incubator for 2 h. After another plate washing, substrate solution (100 μl) was added to each well, and the samples were incubated in the dark for 30 min. Finally, the reaction was stopped with 100 μl of stop solution, and OPG concentrations were measured at a wavelength of 450 nm.

### Tartrate-resistant acid phosphatase (TRAP) staining

Using a TRAP Kit (Nanjing Jiancheng Bioengineering Institute, Nanjing, China), we fixed the cells with fixation fluid at room temperature for 30 s, after which the cells were washed with deionized water. The TRAP-stained liquor was prepared gradually. After being dewaxed, the paraffin sections were stained in preheated stained liquor for 1 h at 37°C, rinsed with distilled water, stained with Fast Green for 1 min, dehydrated in an ethanol gradient, cleared with xylene, and sealed using neutral gum. The results were observed under an inverted microscope. TRAP-positive cells exhibited red-stained cytoplasm, while control cells exhibited yellow staining. After being redyed with hematoxylin, cell nuclei exhibited blue staining, while the cytoplasm exhibited red or weak red staining.

### Cell culture

After recovery, THP-1 cells (provided by the cell bank of the Institute of Biochemistry and Cell Biology, Shanghai Institutes for Biological Sciences, Chinese Academy of Sciences, Shanghai, China) were cultured in RPMI-1640 medium containing 10% inactivated fetal bovine serum, penicillin and streptomycin antibiotics at 37°C in 5% CO_2_, and HEK-293T cells were cultured in DMEM containing 10% fetal bovine serum, penicillin and streptomycin antibiotics [27].

### Luciferase reporter gene assay

The 3'-untranslated region (UTR) of *OPG* was joined to a luciferase reporter vector, which was co-transfected into THP-1 cells with an miR-145 precursor (pre-miR-145). According to the instructions of the Dual-Luciferase Reporter Assay System (Promega, Madison, WI, USA), the cells were disrupted to detect biological luminescence and to observe the targeting of OPG by miR-145. Twenty-four hours before transfection, THP-1 cells in good condition were seeded into 6-well plates at a density of 30 × 10^4^ cells per well. Transfection was conducted at a cell density of 80%. Plasmid DNA and Lipo2000 diluted in Opti-MEM (250 ul) were mixed well at room temperature. After standing for 25 min, this mixture was added to corresponding 6-well plates, and cells were collected after being transfected for 48 h. The cells were divided into a nonsense sequence group (non-transfected group) and an miR-245 mimic group (transfected group).

### Reverse transcription-polymerase chain reaction (RT-PCR)

mRNA was extracted from THP-1 cells via the Trizol method [28] and then reverse-transcribed into cDNA via PCR (Bio-Rad Reverse Transcription kit; Bio-Rad Laboratories, Richmond, CA) under the following reaction conditions: 25°C for 5 min, 42°C for 30 min, and 85°C for 5 min. An ABI7500 quantitative PCR instrument (Applied Biosystems) was used for RT-PCR analysis. Primer 5.0 was used to design the RT-PCR primers (Table 1), and Opticon-Monitor 3 software (Bio-Rad, US) and a fluorescence microscope (Leica, Germany) were used to examine the PCR results. The 2^-ΔΔCt^ method was used for analysis of miR-145, OPG, PANK and PANKL expression. To amplify OPG, PANK, and RANKL, we used a quantitative PCR (q-PCR) reaction system with a total volume of 20 μl comprising 2 μl of cDNA template, 0.4 μl of upstream primer and 0.4 μl of downstream primer for OPG, PANK and RANKL, respectively, as well as 10 μl of 2X SYBR Green SuperMix. PCR amplification was performed as follows: annealing at 57°C for 30 s, followed by extension at 72°C for 45 s and denaturation at 94°C for 30 s for a total of 30 cycles, as well as a final reaction step at 72°C for 10 min. U6 and β-actin served as internal controls.

**Table 1:** Primers used in reverse transcription-polymerase chain reaction.

Gene symbol	Forward primer	Reverse primer
	(5’-3’)	(5’-3’)
**miR-145**	GTCCAGTTTTCCCAGGAATCCCT	TGGTGTCGTGGAGTCG
**U6**	GTTGCGTTACACCCTTTCTTG	GTCACCTTCACCGTTCCAGT
**OPG**	ACAATGAACAAGTGGCTGTGCTG	CGGTTTCTGGGTCATAATGCAAG
**PANK**	GGCTGGCTACCACTGGAACT	TCCTGTAGTAAACGCCGAAGA
**RANKL**	GCAGCATCGCTCTGTTCCTGTA	GCATGAGTCAGGTAGTGCTTCTGTG
**β-actin**	AGACCACCTTCAACTCGATCAT	ACTCGTCATACTCCTGCTTGCT

miR-145, microRNA-145; RANKL, receptor activator of nuclear factor-κ B ligand; OPG, osteoprotegerin; RANK, receptor activator of nuclear factor-κ B; U6 and β-actin, internal controls.

### Western blot assay

After being transfected with miR-145, 293T cells and THP-1 cells were collected at 24 h, 48 h and 72 h. Total protein was extracted after cell disruption, and protein concentrations were estimated with the bicinchoninic acid (BCA) method. Then, the proteins were separated by 12% sodium dodecyl sulfate-polyacrylamide gel electrophoresis (SDS-PAGE), in which gel separation was performed at 80 V, and gel concentration was performed at 120 V, and subsequently transferred to polyvinylidene fluoride (PVDF) membranes using 280 Ma for 100 min. Primary antibodies against OPG (1:500, cat-sc8468, Santa Cruz Biotechnology, Inc., Santa Cruz, CA, USA) and RANKL (1:1,000, 12055, CST Biological Reagents Company Ltd., Shanghai, China) were added to the membranes, which were incubated at 4°C overnight. After the membranes were rinsed with Tris-buffered saline with Tween-20 (TBST), the appropriate secondary antibodies (Boster Company, Wuhan, China) were added. After development, protein expression was detected.

### Statistical analysis

SPSS 19.0 software (SPSS Inc., Chicago, IL, USA) was used for statistical analysis. Measurement data are presented as the mean ± standard deviation (SD) and were tested for normality of distribution. The *t* test was applied for comparisons between two groups, and one-way analysis of variance (ANOVA) was used for comparisons among multiple groups, before which the homogeneity-of-variance test was performed. Pairwise comparisons of mean values were conducted via the least significant difference test (LSD-t). *P* values less than 0.05 were considered statistically significant.

## Results

### Lentiviral vector titer determination

**Fig 1:**
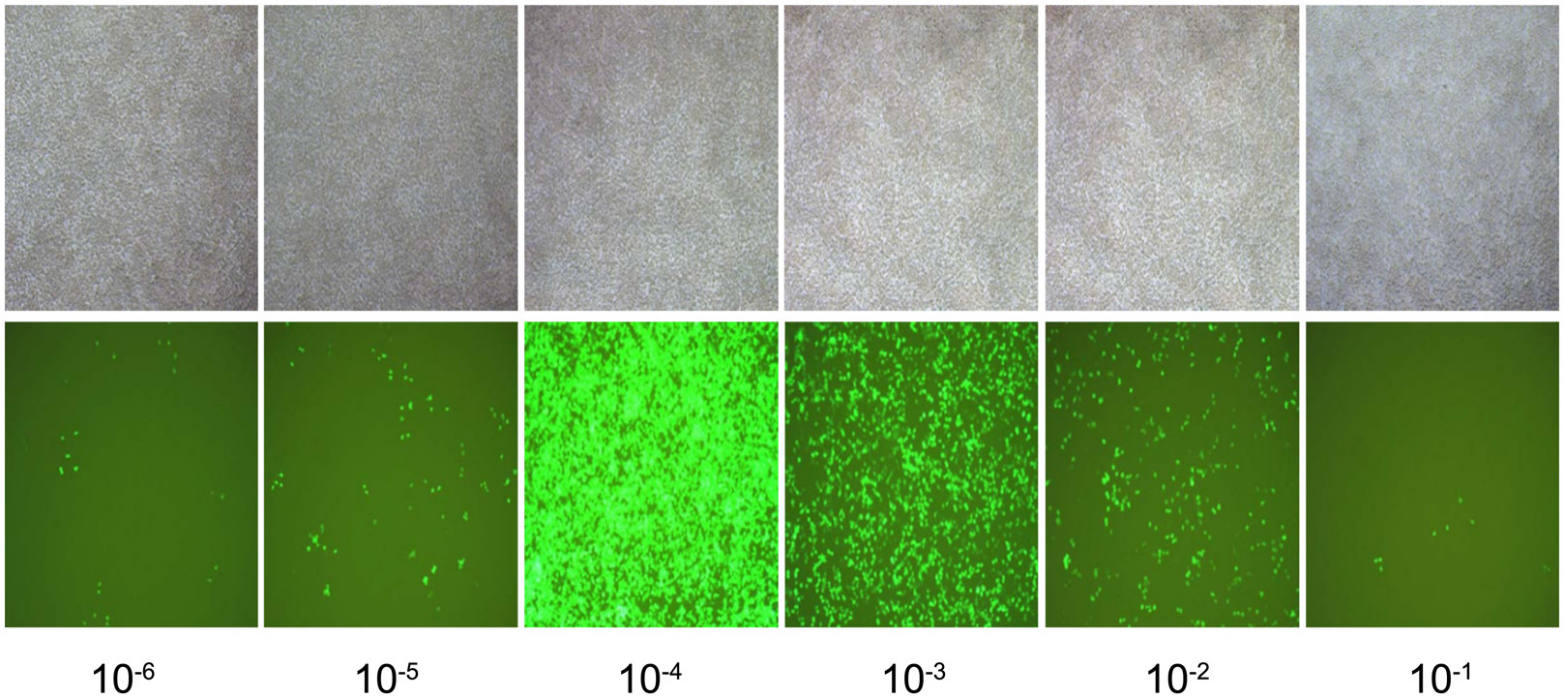
Fluorescence detection in 293T cells at 48 h after plasmid transfection. Note: the experiment was repeated 3 times, and means were obtained.

After 293T cells were transfected with the miR-145 overexpression vector for 48 h, GFP fluorescence intensity was observed using a fluorescence microscope to determine transfection efficiency. As seen in Fig 1, transfection efficiency gradually increased with increasing viral titersand peaked at a viral titer of 10^−4^.

### MiR-145 and OPG expression in 293T cells after transfection with the miR-145 overexpression vector

**Fig 2:**
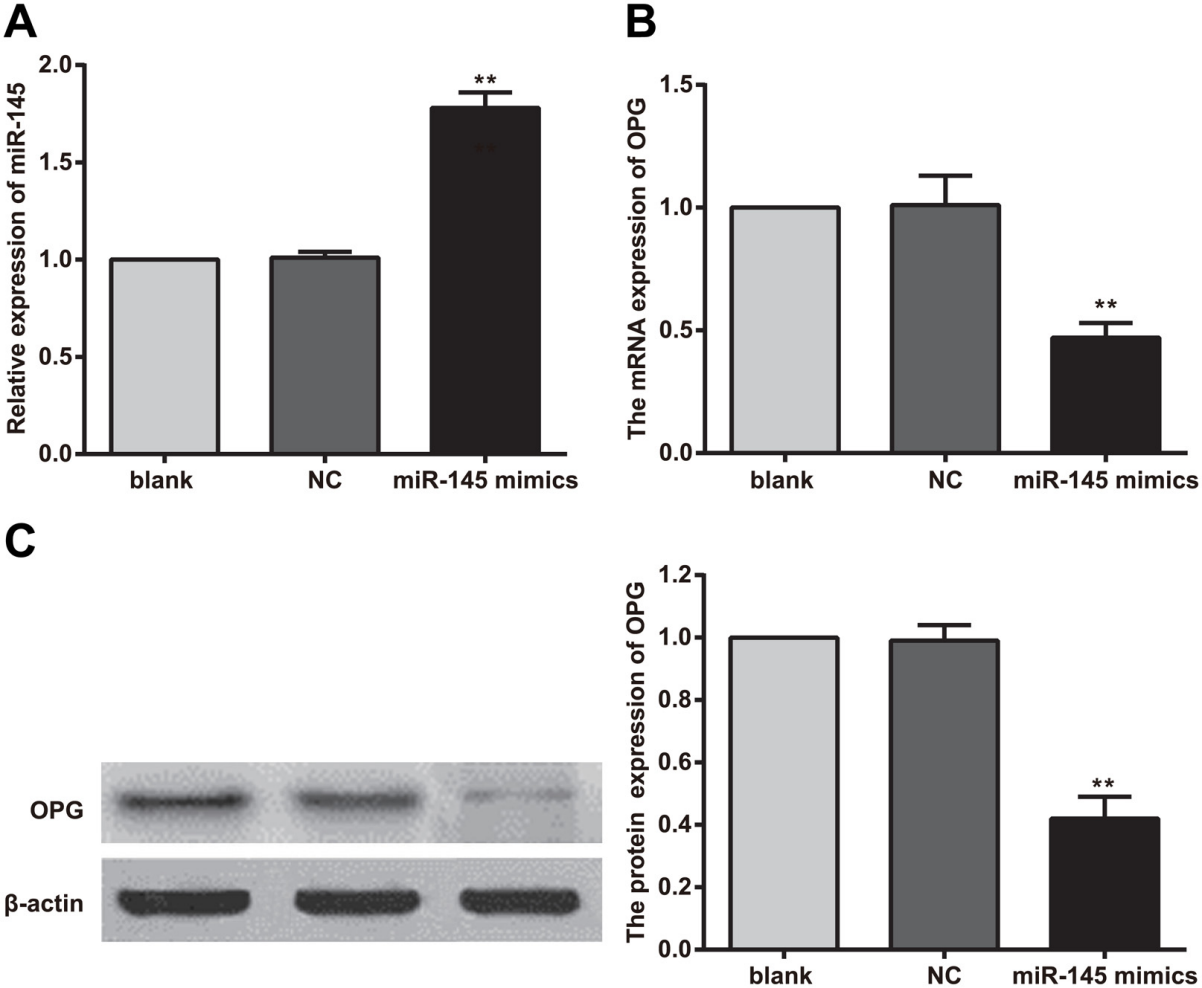
Changes in microRNA (miR)-145 and osteoprotegerin (OPG) expression in 293T cells after transfection with the miR-145 overexpression vector. Note: A, miR-145 level after transfection; B, OPG mRNA level after transfection; C, OPG protein level after transfection; **, compared with the control group and normal group, *P* < 0.01; experiments were repeated 3 times, and means were obtained.

After 293T cells were transfected with the miR-145 overexpression vector, the expression level of miR-145 in 293T cells was significantly increased compared with that in control cells and normal cells (both *P* < 0.01). After miR-145 overexpression, the levels of OPG mRNA and protein expression decreased significantly compared with their corresponding levels in the control and normal groups (both *P* < 0.05) (Fig 2).

### MiR-145 and OPG expression in rat serum

**Fig 3:**
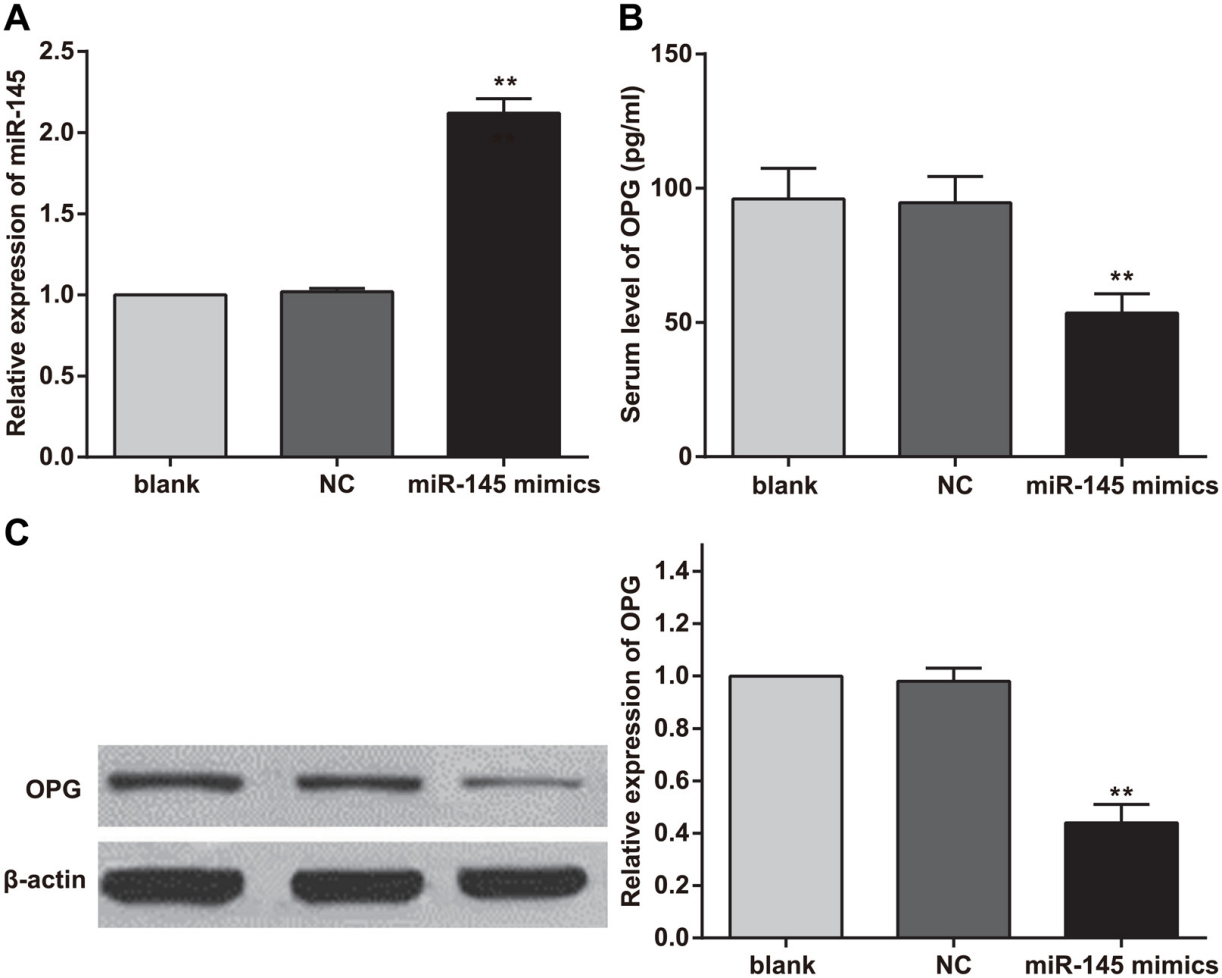
Changes in serum microRNA (miR)-145 and osteoprotegerin (OPG) levels after transfection. Note: A, serum miR-145 level after transfection; B, serum OPG concentration after transfection; C, serum OPG protein expression level after transfection; **, compared with the blank group and negative control (NC) group, *P* < 0.01; experiments were repeated 3 times, and means were obtained.

Lentiviruses containing miR-145 and control sequences were injected into rats with SIFHN via the tail vein, and serum miR-145 expression levels were detected by real-time PCR at 1 week after injection. The results showed that miR-145 expression was significantly up-regulated in the lentivirus-mediated miR-145 group compared with the control and normal groups (both *P* < 0.01). The level of miR-145 expression in the lentivirus-mediated negative control (NC) group was not significantly different from that of the normal group (*P* > 0.05) (Fig 3A). Serum *OPG* concentrations were detected by ELISA, which showed that miR-145 overexpression elicited significant decreases in serum *OPG* levels (*P* < 0.05) (Fig 3B). OPG protein expression levels in the rat model of SIFHN were evaluated by western blotting. Additionally, gray-scale analysis was performed using Image J software (NIH, Bethesda, MD, USA). The results indicated that miR-145 overexpression elicited a decrease in the expression level of OPG protein and that the differences in OPG protein expression between the lentivirus-mediated miR-145 group and lentiviral control and normal groups were significant (both *P* < 0.01). Moreover, there was no difference in OPG protein expression between the lentivirus-mediated NC group and normal group (Fig 3C).

### OPG is a target gene of miR-145

**Fig 4:**
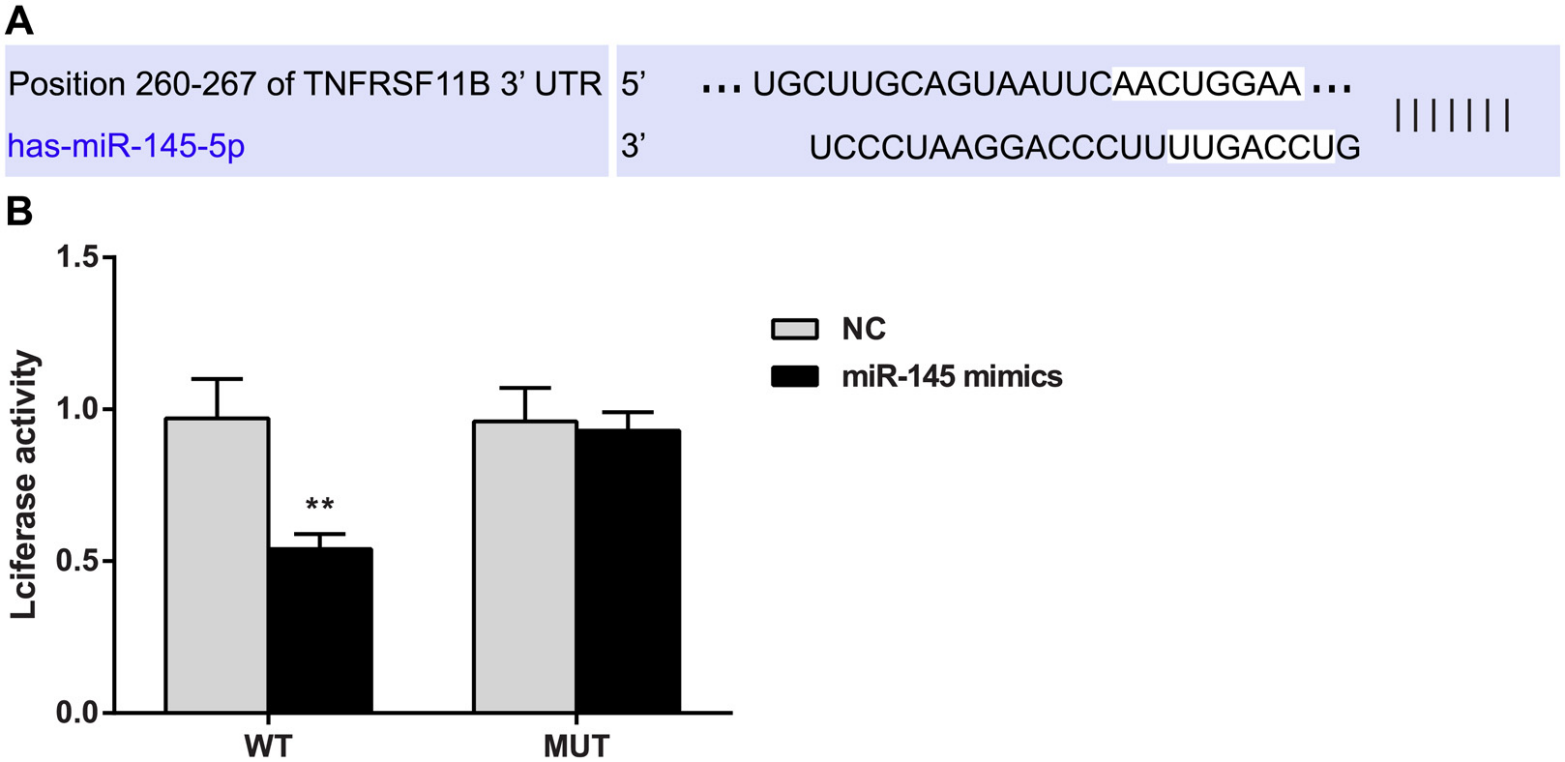
Double-luciferase reporter gene assay confirms that *osteoprotegerin* (*OPG*) is the target gene of microRNA (miR)-145. Note: **, compared with the negative control (NC) group, *P* < 0.01; WT, wild type; MUT, mutation; experiments were repeated 3 times, and means were obtained.

TargetScan (http://www.targetscan.org/), the online miRNA target gene prediction tool, was utilized to predict the target genes of miR-145 and showed that *OPG* (TNFRSF11B) is one of the target genes of miR-145 and that the binding regions of the 3 'UTR of the *OPG* gene and miR-145 are highly conserved in mammals (Fig 4A).

The luciferase reporter gene assay results indicated that relative luciferase activity decreased after co-transfection of the 3'UTR of the wild-type *OPG* gene (*OPG* 3’UTR-WT) and the miR-145 mimic (*P* < 0.01), while no changes in relative luciferase activity occurred after co-transfection of the 3'UTR of the mutant *OPG* gene (OPG 3’UTR-MUT) and the miR-145 mimic (Fig 4B).

### Pathological observation of bone necrosis

**Fig 5:**
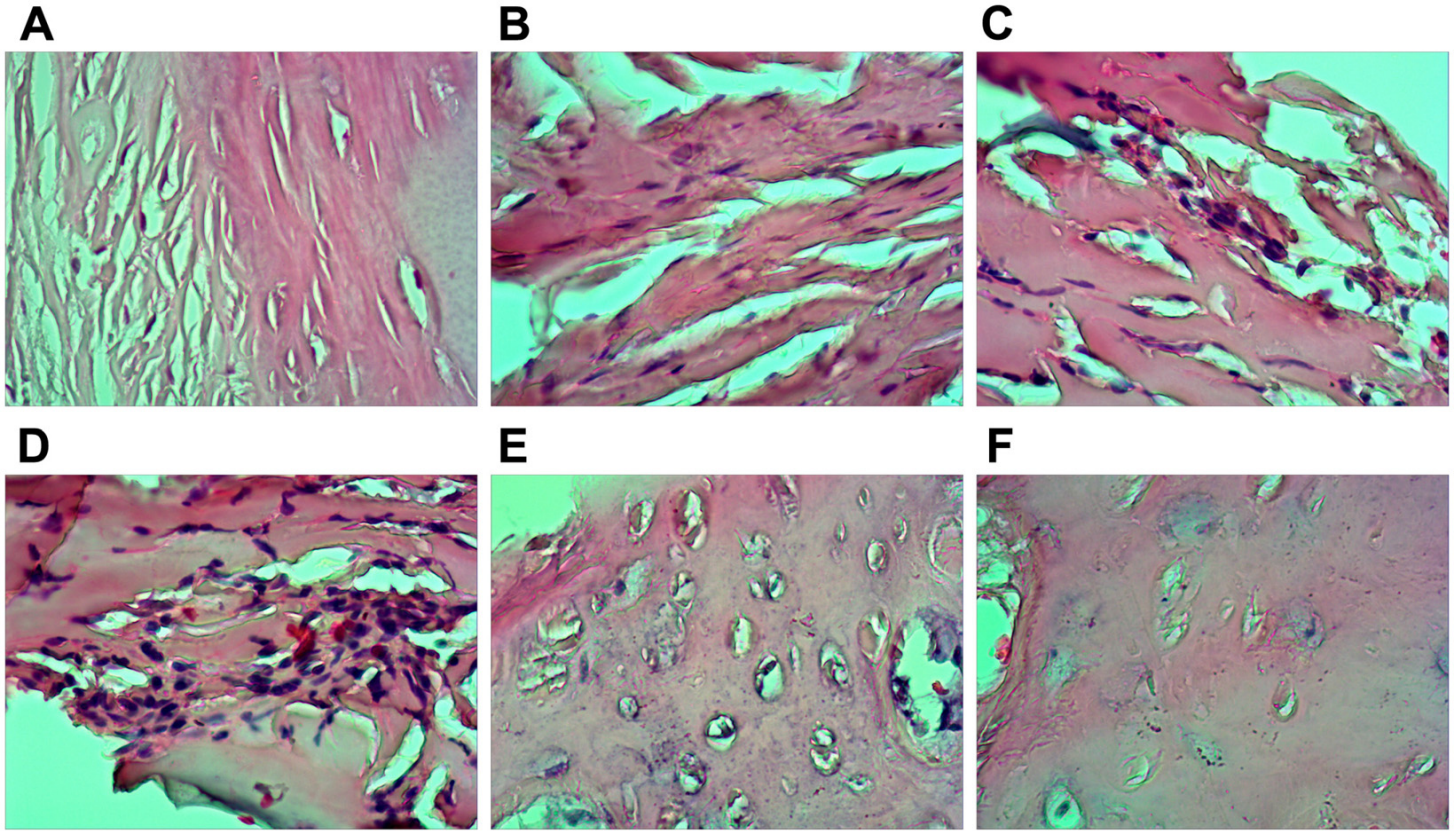
Histopathological features of necrotic femoral head tissues by hematoxylin eosin (HE) staining. Note: A. arrangement of trabecular bone in the control group; B. arrangement of trabecular bone at the 6^th^ week of treatment in the experimental group; C. arrangement of trabecular bone at the 8^th^ week of treatment in the experiment group; D. arrangement of trabecular bone at the 10^th^ week of treatment in the experiment group; E. lacunae in trabecular bone in the control group; F. lacunae in trabecular bone in the experimental group; experiments were repeated 3 times.

**Fig 6:**
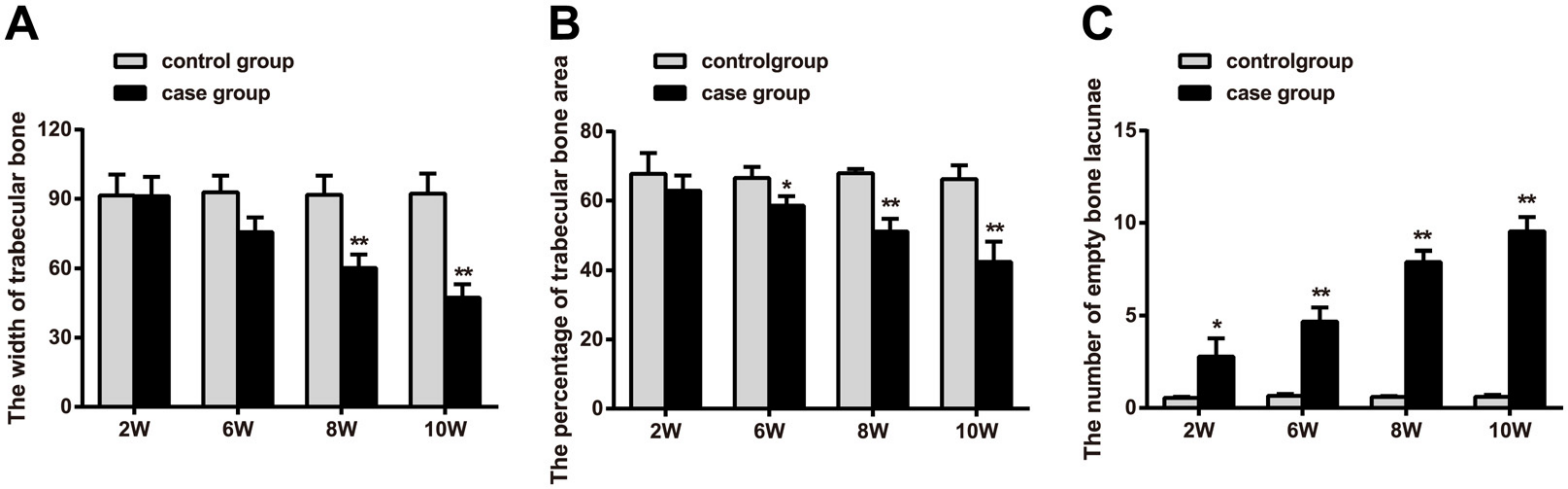
Changes in caput humeri parameters in the experimental group and control group. Note: A, trabecular bone width measurement results in the control group and experimental group; B, percentage of trabecular bone area in the control group and experimental group; C, numbers of empty bone lacunae in the control group and experimental group by the *t* test; *, compared with the control group, *P* < 0.05; **, compared with the control group, *P* < 0.01.

Areas of necrotic bone were subjected to hematoxylin-eosin (HE) staining, and the pathological characteristics of these lesions were observed. The staining results (Fig 5) indicated that after 2 weeks of treatment with intramuscular dexamethasone, significantly enhanced lacunar wall structures, as well as pyknosis, deep dyeing, and empty bone lacunae, were visible on one side of the bone cell nucleus. After six weeks of treatment, trabecular bone thinning and structural irregularities were noted. After eight weeks of treatment, significantly enlarged fat cells were noted in the bone marrow, including cells that had grown large enough to fuse with adjacent cells, resulting in larger amounts of free bone tissue. After 10 weeks of treatment, increased numbers of fractures were visible, as were significantly increased numbers of enlarged fat cells in the bone marrow. HE staining showed that intramuscular injections of dexamethasone sodium phosphate can be used to induce femoral head necrosis in SD rats. As shown in Fig 6, the trabecular bone width of the femoral head was significantly smaller in the experimental group than in the control group (*P* < 0.05) after six weeks of treatment, which was consistent with the HE staining results. Beginning with the sixth week of treatment, the percentage of trabecular bone area in the experimental group gradually increased compared with the control group (*P* < 0.05). Beginning during the second week of treatment, the number of empty lacunae noted under high magnification in the experimental group was significantly greater than that noted in the control group, a difference that was statistically significant (*P* < 0.05).

### TRAP staining

**Fig 7:**
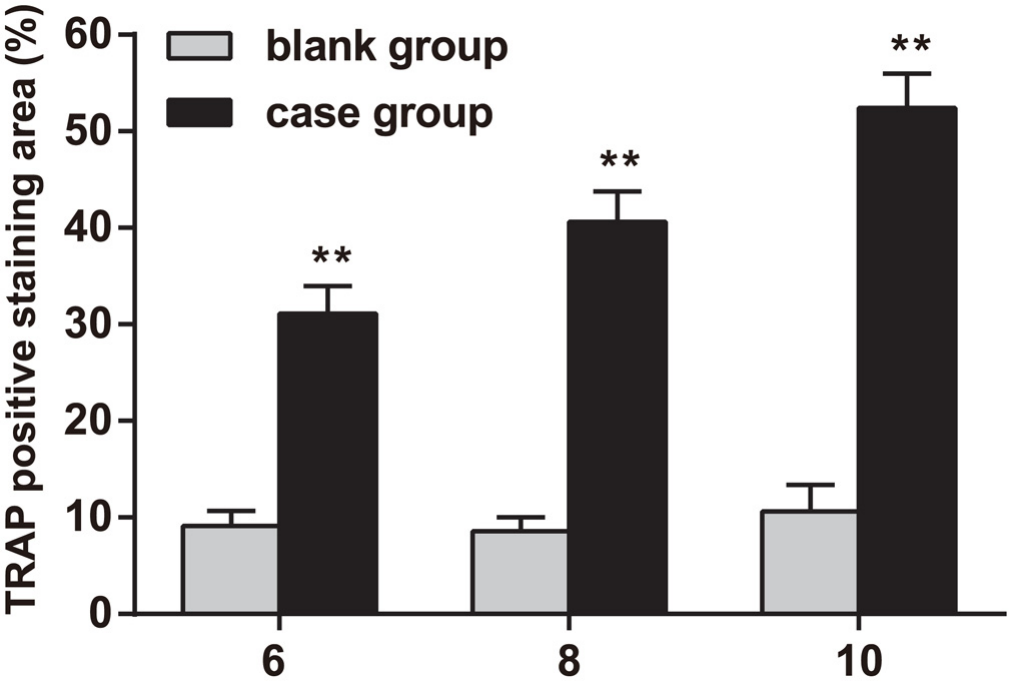
Tartrate-resistant acid phosphatase (TRAP) staining in the rat model of steroid-induced necrosis of the femoral head at the 6^th^, 8^th^ and 10^th^ weeks of treatment. Note: **, compared with the control group, *P* < 0.01; experiments were repeated 3 times, and means were obtained.

TRAP staining was visible in the bone marrow and on the bone trabecular surfaces of the lower cartilage zone (as seen in Fig 7). The rate of positive TRAP staining was significantly higher in the experimental groupthan in the normal control group after six weeks of treatment, and the rate of positive TRAP staining was 80% higher in the experimental group than in the normal control group after ten weeks of treatment (*P* < 0.01).

### OPG, RANK and RANKL mRNA expression in THP-1 cells at different time points

**Fig 8:**
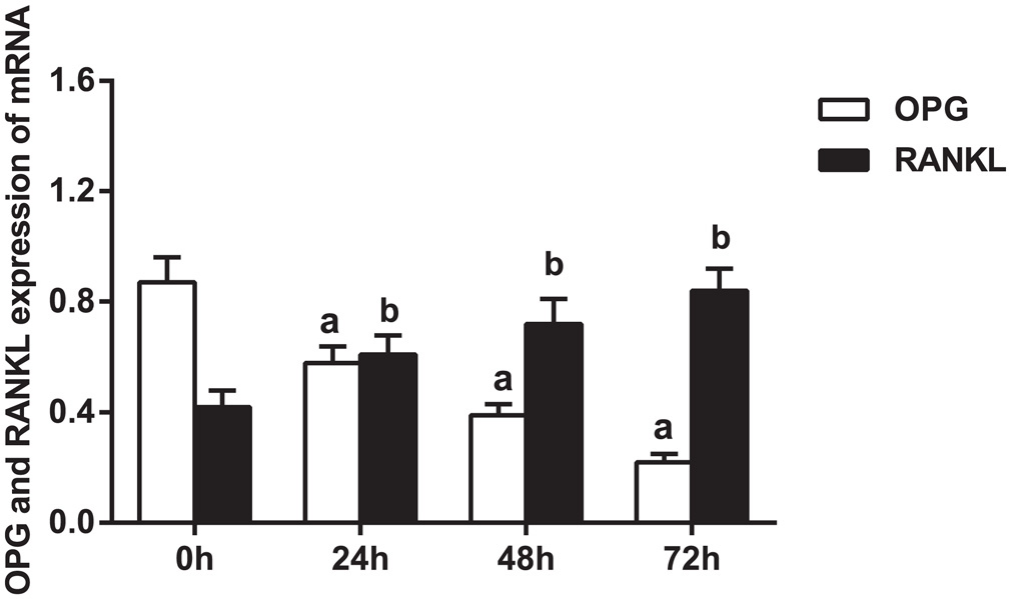
mRNA expression levels of OPG, RANKL and RANK after microRNA (miR)-145 overexpression at different time points. Note: a, compared to 0 h, *P* < 0.01; b, compared to 0 h, *P* < 0.01; RANKL, receptor activator of nuclear factor-κ B ligand; OPG, osteoprotegerin; RANK, receptor activator of nuclear factor-κ B; experiments were repeated 3 times, and means were obtained.

MiR-145 was transfected into THP-1 cells, and the gene expression levels of RANKL, RANK and OPG were subsequently determined via real-time PCR in the experimental group, which overexpressed miR-145. As shown in Fig 8, transfected cells were collected for total RNA extraction for gene detection after 24, 48, and 72 h. The results indicated that the mRNA expression level of OPG gradually decreased, while the mRNA expression levels of RANK and RANKL significantly increased in the setting of miR-145 overexpression.

### OPG, PANK and PANKL protein expression in THP-1 cells at different time points

**Fig 9:**
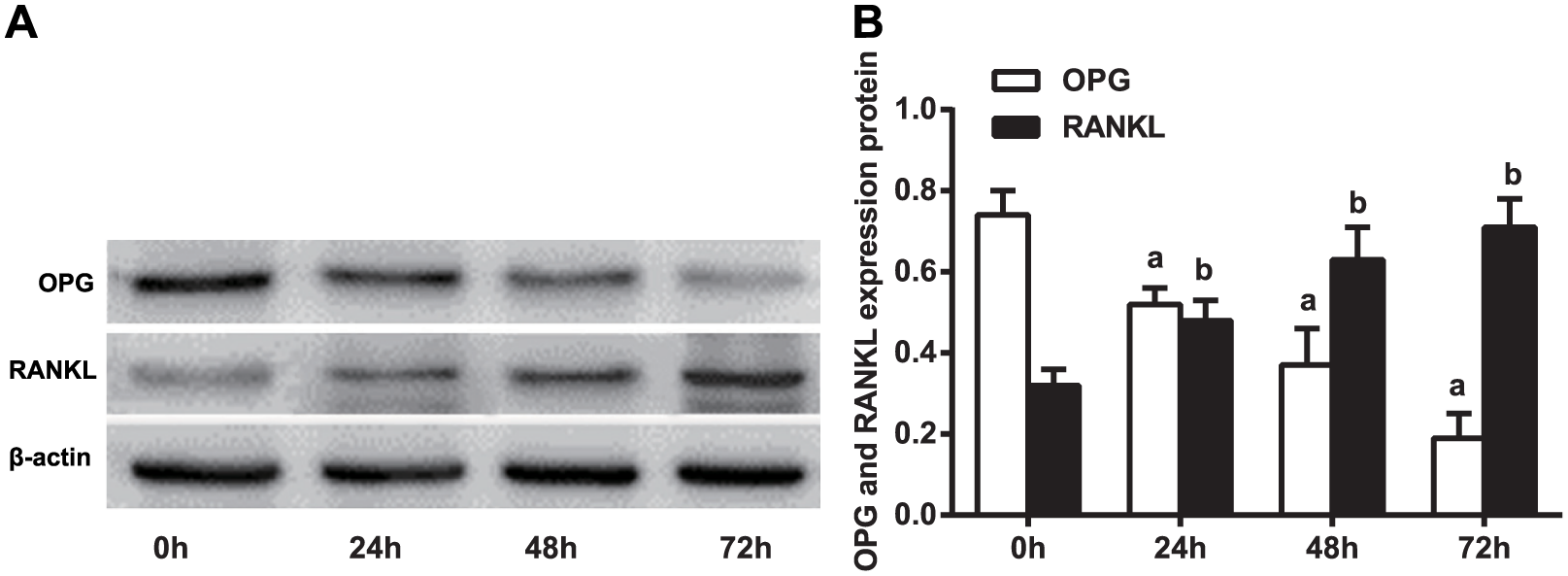
Protein expression levels of OPG, RANKL and RANK after microRNA (miR)-145 overexpression at different time points. Note: a, compared to 0 h, *P* < 0.01; b, compared to 0 h, *P* < 0.05; RANKL, receptor activator of nuclear factor-κ B ligand; OPG, osteoprotegerin; RANK, receptor activator of nuclear factor-κ B; experiments were repeated 3 times, and means were obtained.

The levels of OPG, RANK and RANKL protein expression were detected by western blotting. As shown in Fig 9, OPG protein content decreased in a time-dependent manner after miR-145 transfection. Gray value analysis indicated that OPG protein content had significantly decreased by 1.4 fold at 72 h after transfection compared to its baseline value at 0 h. The protein expression levels of RANK and RANKL increased in a time-dependent manner after transfection and peaked at 72 h after transfection.

## Discussion

Due to the complexity of the pathogenesis of SINFH, new therapies for this disease have been extensively investigated. Trials exploring the effects of endogenous miRNAs on specific diseases may be helpful in identifying therapeutic targets to facilitate treatment [22]. However, target miRNA identification is a significant obstacle in this endeavor. Previous studies have shown that miR-145 is downregulated in hormone-NO patients using miRNA chip assays; thus, we focused on miR-145 in the present study and performed a series of experiments to determine its role in SINFH and the mechanisms underlying its effects [17–19].

In the present study, we successfully constructed a rat model of SINFH via tail vein injections of a lentiviral vector, pLV-shRNA-miR-145, and observed that miR-145 expression increased markedly in SINFH rats compared with control rats. By acting as a decoy receptor for RANKL and preventing RANKL from binding to RANK, OPG inhibits osteoclast differentiation and prevents excessive bone resorption [29]. ELISA and western blotting were conducted to measure serum OPG levels in SINFH rats and showed that OPG protein levels were decreased in the setting of miR-145 overexpression, suggesting that miR-145 expression may be negatively correlated with OPG levels in SINFH. Consistent with our results, Wang *et al*. demonstrated that OPG expression levels were markedly decreased in a glucocorticoid group receiving twice-weekly intramuscular injections of 12.5 mg of prednisolone [25]. Furthermore, *OPG* was confirmed as an miR-145 target gene using TargetScan. Although the exact relationship among miR-145, OPG and SINFH is unknown, RANKL and RANK are believed to be involved in the mechanisms underlying the effects of miR-145 on OPG levels and function in SINFH.

Additionally, we evaluated OPG, RANKL and RANK mRNA and protein expression in THP-1 cells treated with miR-145 transfection using real-time PCR and western blotting. We found that the mRNA and protein expression levels of OPG decreased, while those of RANKL increased, suggesting that miR-145 overexpression may lead to decreased OPG expression and increased RANKL expression, thereby playing a key role in the development and regeneration of bone cells in SINFH. The pathogenesis of SINFH is characterized by disordered bone metabolism caused by an imbalance between the anabolic and catabolic actions of osteoclasts [30]. MiRNAs are believed to be key regulators of osteoclast-mediated bone resorption, as well as osteoblast proliferation and differentiation [31]. In this study, we characterized miR-145, a novel miRNA associated with SINFH, and found that its effects on osteoblast differentiation in SINFH involve the OPG/RANK/RANKL pathway. OPG is a soluble receptor that inhibits osteoclastogenesis by binding to RANKL and suppressing the interaction between RANKL and RANK that is required for osteoclastogenesis stimulation, as shown in *in vivo* studies. The binding of RANKL to RANK activates signaling cascades that control the lineage commitments and activity of osteoclasts [32]. Consistent with our findings, it has been shown that the expression level of OPG mRNA was significantly lower, and the expression level of RANKL was significantly higher in SD rats treated with glucocorticoids than in control rats. Moreover, the OPG/RANKL ratio was significantly lowerin treated rats than in control rats in the study in question [25]. In addition, Samara *et al*. found that differential expression of OPG, RANK and RANKL can disturb bone homeostasis and triggerbone destruction and subsequent collapse of the hip joint [32].

In conclusion, the present study has provided evidence that miR-145 regulates osteoblast proliferation and regeneration in SINFH by targeting the OPG/PANK/PANKL signaling pathway. Our findings have provided us with new insights regarding the roles of miRNAs in SINFH and may facilitate the development of new therapies for SINFH. We should mention that the relatively small number of subjects included in this study may have affected the significance of our results due to low statistical power; however, the rats used in this study were in good condition and did not exhibit significant morbidity and mortality during the experiment. The final data sets comprised mean values from each group; therefore, these data were reliable. In addition, due to time and budget constraints, our experiments were not repeated. Time and budget permitting, we will repeat our experiments to verify our results and will carry out a more in-depth study in the future.
